# Lipreading Architecture Based on Multiple Convolutional Neural Networks for Sentence-Level Visual Speech Recognition

**DOI:** 10.3390/s22010072

**Published:** 2021-12-23

**Authors:** Sanghun Jeon, Ahmed Elsharkawy, Mun Sang Kim

**Affiliations:** Center for Healthcare Robotics, Gwangju Institute of Science and Technology (GIST), School of Integrated Technology, Gwangju 61005, Korea; jeon7887@gist.ac.kr (S.J.); elsharkawy@gm.gist.ac.kr (A.E.)

**Keywords:** 3D densely connected CNN, 3D multi-layer feature fusion CNN, convolutional neural network, deep learning, lipreading, speech recognition, visual speech recognition

## Abstract

In visual speech recognition (VSR), speech is transcribed using only visual information to interpret tongue and teeth movements. Recently, deep learning has shown outstanding performance in VSR, with accuracy exceeding that of lipreaders on benchmark datasets. However, several problems still exist when using VSR systems. A major challenge is the distinction of words with similar pronunciation, called homophones; these lead to word ambiguity. Another technical limitation of traditional VSR systems is that visual information does not provide sufficient data for learning words such as “a”, “an”, “eight”, and “bin” because their lengths are shorter than 0.02 s. This report proposes a novel lipreading architecture that combines three different convolutional neural networks (CNNs; a 3D CNN, a densely connected 3D CNN, and a multi-layer feature fusion 3D CNN), which are followed by a two-layer bi-directional gated recurrent unit. The entire network was trained using connectionist temporal classification. The results of the standard automatic speech recognition evaluation metrics show that the proposed architecture reduced the character and word error rates of the baseline model by 5.681% and 11.282%, respectively, for the unseen-speaker dataset. Our proposed architecture exhibits improved performance even when visual ambiguity arises, thereby increasing VSR reliability for practical applications.

## 1. Introduction

Speech is the most common form of communication between humans and involves the perception of both acoustic and visual information. In 1976, McGurk and McDonald demonstrated that speech perception is influenced by vision, which is called the McGurk effect [1]. This effect indicates the necessity of matching both auditory and visual phonemes to perceive pronounced phonemes correctly.

Vision plays a crucial role in speech understanding, and the importance of utilizing visual information to improve the performance and robustness of speech recognition has been demonstrated [2,3,4]. Although acoustic information is richer than visual information when speaking, most people rely on watching lip movements to fully understand speech [2]. Furthermore, people rely on visual information in noisy environments where receiving auditory information is challenging. Similarly, people with hearing impairments depend on visual information to perceive spoken words. However, comprehending oral language using visual information alone, especially in the absence of context, can be challenging because it is difficult to understand lipreading actuations such as lip, tongue, and teeth movements without context [3]. Hearing-impaired people using visual information have achieved an accuracy of 17 ± 12%, even for a small subset of 30 monosyllabic words, and 21 ± 11% for 30 compound words, according to Easton and Basala [4]. Chung et al. [5] showed that experienced professional lip-leaders achieved 26.2% accuracy with the BBC News benchmark dataset when they could watch an unlimited number of videos.

The development of a visual speech recognition (VSR) system has enormous potential for various practical applications, such as speech recognition in noisy environments, biometric identification for security, communication in underwater environments, and silent movie analysis, and it can positively affect patients with speech impairments [6,7]. Therefore, it is important to develop a VSR system that exclusively uses visual information.

Recently, several researchers have investigated the possibility of developing a VSR system by decoding speech using only visual information to mimic human lipreading capability [8,9,10,11,12,13]. Despite their efforts, VSR systems still exhibit low performance and accuracy compared to those of audio or audio-VSR systems. A major challenge is the distinction of words with similar pronunciation, called homophones [9]; these lead to ambiguity at the word level. For example, although some words, such as pack, back, and mac, differ in their sound, the characters (e.g., [p], [b], and [m]) produce almost identical lip movements, thereby making them difficult to distinguish. As such, word distinction is the most difficult task for humans and crucial for accurate lipreading [14]. Another technical limitation of traditional VSR systems is that visual information does not provide sufficient data for learning words such as “a”, “an”, “eight”, and “bin” because their length is no longer than 0.02 s [15].

To address the challenges of similar pronunciation and insufficient visual information, this paper presents a novel lipreading architecture that exhibits superior performance compared to those of traditional and existing deep learning VSR systems. This architecture consists of two sub-networks using end-to-end neural networks: the visual feature extraction module is made of a 3D convolutional neural network (CNN), a 3D densely connected CNN for each time step by reducing model parameters, and a multi-layer feature fusion (MLFF) CNN for capturing multichannel information in the temporal dimension of the entire video and localizing effective objects. The sequence processing module uses a two-layer bi-directional gated recurrent unit (GRU), which is followed by a linear layer. After applying a SoftMax layer to all time steps to obtain the probabilities, the entire network is trained using the connectionist temporal classification (CTC) loss function.

In our experiment, we compared the accuracy and efficiency of our architecture with those of other visual feature extraction models with excellent performance on a benchmark dataset [16]. The models used for this comparison were LeNet-5 [17], VGG-F [18], ResNet-50 [19], DenseNet-121 [20], and LipNet [21] as the baseline model. Extensive evaluation results show that the proposed architecture achieves state-of-the-art results and remarkable efficiency compared to existing deep learning methods.

The contributions of our work can be summarized as follows:

- We developed a novel lipreading architecture based on end-to-end neural networks that relies exclusively on visual information;
- We compared the architecture of our proposed model with that of LipNet as the baseline and those of 3D LeNet-5, 3D VGG-F, 3D ResNet-50, and 3D DenseNet-121 to evaluate the reliability of our model for practical applications;
- We demonstrated improved accuracy and efficiency of the proposed architecture over existing deep learning architectures applied to VSR system implementation.

The remainder of this paper is organized as follows. Section 2 reviews related work on VSR systems and the traditional and existing deep learning approaches. Section 3 introduces the proposed architecture. Section 4 presents information on benchmark datasets, data processing, data augmentation, implementation, and performance evaluation metrics. Along with certain comparative experiments and public processes, this section presents the experimental results. Finally, Section 5 provides a discussion and our conclusions.

## 2. Related Work

**Figure 1:**
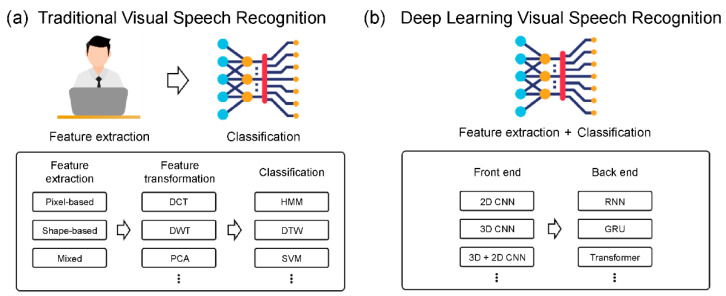
VSR process: (**a**) traditional step and (**b**) deep-learning step.

This section summarizes the traditional and existing deep learning approaches for VSR systems. Figure 1 illustrates the VSR processes of the traditional and deep-learning-based methods.

### 2.1. Traditional VSR

There are various traditional approaches for implementing VSR systems, such as pre-processing of extracted image features and temporal video feature detection, involving tasks such as optical flow, movement detection, and hand-worked vision pipelines. The traditional approach to implementing the VSR method can be split into two phases. The first phase involves the extraction of visual features from lip movements, and this process relies primarily on hand-labeled features of the geometric information of the lip, such as the lip contour. The existing visual feature extraction method for lip movement involves four steps, starting with the detection and extraction of the region of interest (ROI), the lip, from the video stream, followed by the extraction of lip features from the ROI. For reducing the dimension of the extracted features, a visual feature transformation is required as a complementary step during feature extraction. Different feature transformation algorithms have been developed and used for lipreading tasks, such as linear feature transformation (e.g., discrete cosine transformation (DCT) and discrete wavelet transform (DWT) [22]) and geometry-, motion- [23], and statistical model-based feature transformations [24,25]. The quality of these algorithms depends on the accuracy of training data that are hand-labeled, a task that requires significant amounts of time and effort.

The second phase involves text prediction using the dynamic visual features (classifier phase) and prediction of the words or sentences using a dynamic classifier such as the hidden Markov model (HMM). Using a limited dataset and the HMM model, Goldschen et al. [26] were the first to propose a visual-only sentence-level lipreading technique. They extracted visual features of the mouth region from codebook images to predict continuous sequences of tri-visemes. This study was followed by the development of multi-stream HMMs [27] and the creation of expanded datasets such as model audio and visual streams [22].

As the databases become increasingly complicated, issues such as a high number of speakers, variations in posture, and alterations in the conditions of lighting and background environment may arise. In addition, databases may possess other limitations such as high feature dimension and variations in image quality. Consequently, a complex lip feature extraction algorithm is required. Some classifiers run based on the conditional assumption and are not ideal for modeling long-term dependencies or for operating general classification tasks where several variables are merged.

### 2.2. Deep Learning VSR

In recent years, deep learning methods have been successfully applied to many fields, including VSR systems. Unlike traditional approaches, in which predictions are limited, deep learning methods attain high accuracy. For instance, when a CNN is combined with traditional methods, the trained classifier CNN architecture can distinguish between visemes, and an HMM framework is used to add temporal information after the CNN output [24,25]. Other researchers have combined long short-term memory (LSTM) with histograms of oriented gradients (HoGs) and input recognized short phrases from the GRID dataset [13,16]. Similarly, using the OuluVS and AVLetters datasets, a trained LSTM classifier with DCT and deep bottleneck features was employed to make word predictions [24].

The deep speech recognition architecture that reads the entire input sequence and then predicts the output sentence is called the sequence-to-sequence model (seq2seq). This model uses global information for longer sequences. Watch, listen, attend, and spell (WLAS) was the first seq2seq model to consist of both audio and visual modules, and it was used to recognize audio-visual speech from a real-world dataset [14,28].

The first suggested end-to-end model to deal with sentence-level lipreading and predict character sequences was LipNet [21]. This model combined spatial-temporal convolutions with Bi-GRU and was trained using the CTC loss function. A limited grammar and vocabulary dataset (GRID corpus) was used to evaluate the performance of the LipNet architecture: the word error rates in the overlapped and unseen-speaker databases were 4.8% and 11.4%, respectively, whereas the success rate of human lipreaders for the same database was 47.7%. Similar architectures have been introduced to investigate the convergence of audio-visual features, where digit sequences were predicted using a small subset of 18 phonemes and 11 terms, and a CTC cascading model was used [29,30,31]. Thus, the deep learning method can learn more deeply and extract more comprehensive features from the experimental data, demonstrating strong robustness for big data and visual ambiguity.

## 3. Architecture

**Figure 2:**
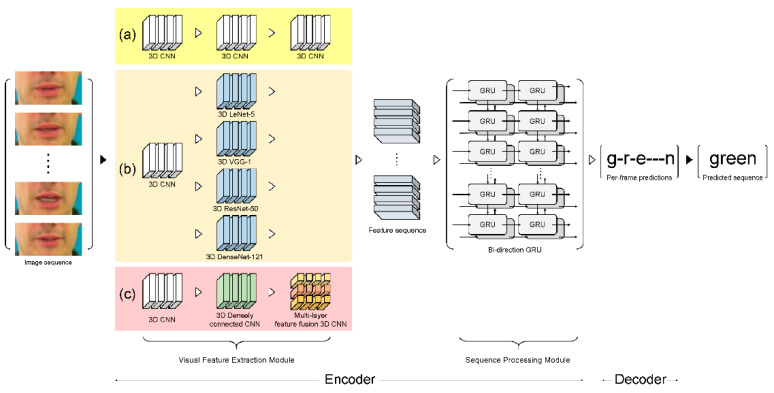
Schematic design of VSR architecture: (**a**) LipNet architecture (baseline), (**b**) four compared architectures, and (**c**) proposed architecture.

**Figure 3:**
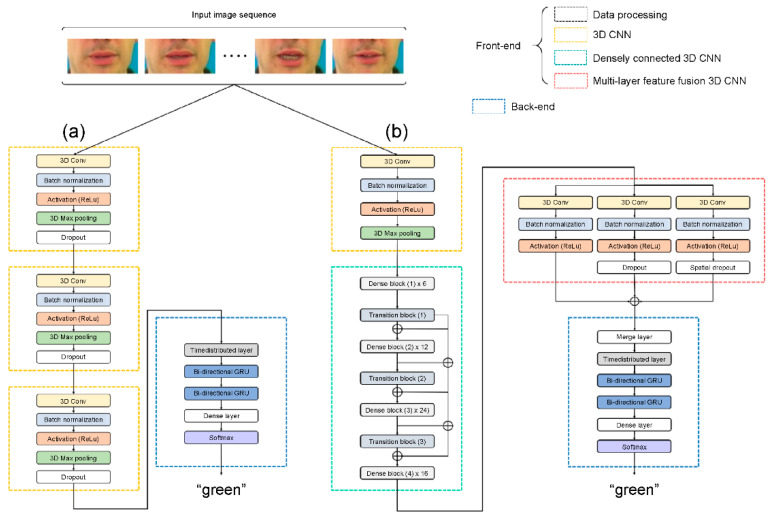
Detailed architecture: (**a**) baseline and (**b**) proposed architecture.

This section describes a VSR deep learning architecture and proposes a novel visual feature extraction module (Figure 2c). The proposed module is compared with other visual feature extraction modules that exhibit outstanding feature extraction performance: (i) LipNet as the baseline module (Figure 2a) and (ii) four other comparative architectures with different visual feature extraction modules, namely, 3D LeNet-5, 3D VGG-F, 3D ResNet-50, and 3D DenseNet-121 (Figure 2b). Figure 3 and (Appendix A—Table A1) provide the detailed hyperparameters describing the proposed architecture.

### 3.1. Spatial-Temporal CNN

**Figure 4:**
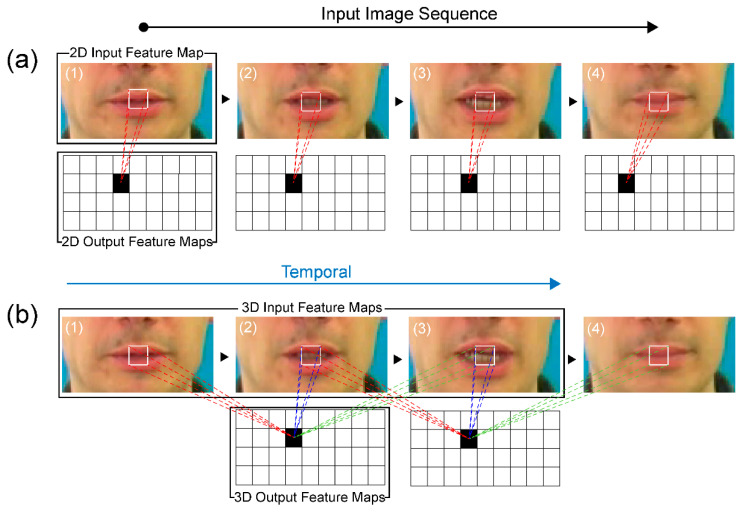
Comparison of (**a**) 2D and (**b**) 3D convolutions.

CNNs directly use raw input data, thereby automating the feature construction process. When a 2D CNN is applied to an image recognition task, it captures the encoded information for a single image’s data and then transfers the encoded information to compute features from the spatial dimensions using 2D feature maps. However, the application of a 2D CNN to a video identification task, where the motion information is encoded in multiple contiguous frames, is ineffective (Figure 4a). Therefore, we use a 3D CNN, which acts as a spatial-temporal CNN in the convolution process, to compute features of both the spatial and temporal dimensions and to capture different lipreading actuations, such as the movements of the lips, tongue, and teeth. This use of a 3D CNN is supported by studies that have shown that 3D CNNs are effective for the extraction of features from video frames, when spatial and temporal information encoded in subsequent frames is considered (Figure 4b) [21,25].

By transforming a single video frame into a cube by stacking several consecutive frames together, the spatial-temporal convolution uses a 3D kernel. In this construction, the feature maps of the convolutional layer are bound to several consecutive frames of the previous layer, which makes it possible to collect motion information during video analysis. Formally, tanh(·) is the hyperbolic tangent function, $b_{ij}$ is the bias for this feature map, $R_{i}$ is the size of the 3D kernel along the temporal dimension, $w_{ijm}^{pqr}$ is the ${\left(p,q,r\right)}^{th}$ value of the kernel linked to the $m^{th}$ feature map in the previous layer, and the value at position (x, y, z) on the j^th^ feature map in the $i^{th}$ layer is given.

(1) $$v_{ij}^{xyz}=\tan h\left(b_{ij}+\underset{m}{\sum }\overset{P_{i}-1}{\underset{p=0}{\sum }}\overset{Q_{i}-1}{\underset{q=0}{\sum }}\overset{R_{i}-1}{\underset{r=0}{\sum }}w_{ijm}^{pqr}v_{\left(i-1\right)m}^{\left(x+p\right)\left(y+q\right)\left(z+r\right)}\right)$$

The heights and weights of the kernels are given by $P_{i}$ and $Q_{i}$, respectively [25]. As 3D convolutional kernels replicate kernel weights around the entire cube in this construction, only one form of feature can be extracted from the frame cube.

In our experiment, all the input video frames were fed into a spatial-temporal CNN to encode the visual information of the extracted lips. To be precise, we obtained spatial-temporal features using a 3D convolutional layer with 64 3D kernels of size 3 × 7 × 7 in the input image extracted from multiple contiguous frames. We used a batch normalization (BN) layer to decrease the transformation of internal covariates and ReLU to speed up the training process. Then, to decrease the spatial scale of the 3D feature maps, a max-pooling 3D layer was added. Thus, the output shape was observed with 75 × 50 × 13 × 64 tensors for an input sequence of 75 × 100 × 50 × 3 (time/width/height/channel) frames.

### 3.2. 3D Densely Connected CNN

A densely connected CNN creates relationships between various layers of the connection, which helps enable full use of the features, reduces the gradient disappearance problem, and deepens the network. Before the convolution layer, the bottleneck layer reduces the input feature volumes. The multichannel feature volumes are then fused following the bottleneck layer process. As the preceding features remain, the next layer is only applied to a small set of feature volumes. In addition, with the hyperparameter theta regulating the degree of compression, transition layers are included to improve the model compactness further. Adopting a bottleneck layer, transition layer, and smaller growth rate results in a narrower network. This strategy reduces the model parameters, effectively suppresses overfitting, and saves computational power.

Although many researchers have used 2D CNNs and extracted visual information separately [28,29], 3D densely connected CNNs were used by adding temporal dimensions to densely connected convolution kernel and pooling layers. This approach was used because 2D CNNs often require complex pre-processing and post-processing to perform the same tasks. Therefore, we modified the 2D DenseNet-121 architecture into a 3D DenseNet-121 architecture to 35 maintain dense connectivity to enable deep feature extraction, and this architecture fully utilizes the information provided by the spatial-temporal CNN simultaneously. The dense block is a primary structure composed of densely connected composite functions in the 3D DenseNet-121 architecture, consisting of three sequential operations: BN, ReLU, and 3D convolution layers. The transition layers between different dense blocks contain a BN, ReLU, 3D convolution layer, and average 3D pooling layer.

**Figure 5:**
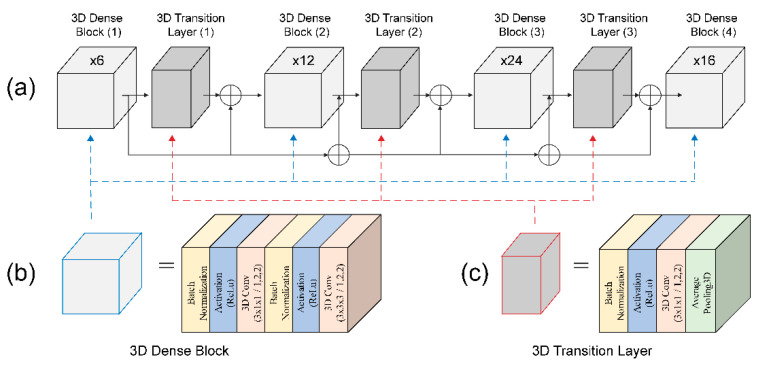
Densely connected 3D CNN architecture: (**a**) detailed densely connected 3D CNN; (**b**) 3D dense block structure; and (**c**) 3D transition layer structure.

We extended the strengths of 2D DenseNet-121 to 3D volumetric image processing tasks. The 2D DenseNet-121 network consists of l layers, where each layer represents a nonlinear transformation $H_{l}$. The output of the lth layer can then be written as $x_{l}$, defined as:

(2) $$x_{l}=H_{l}\left(\left[x_{0},\;x_{1},\;\ldots ,\;x_{1-1}\right]\right)$$

where $x_{0},\;x_{1},\;\ldots ,\;x_{1-1}$ are the volumes of the 3D features produced from the previous layers, and […] refers to the operation of concatenation. Figure 5a illustrates a 3D densely connected CNN architecture consisting of four adjacent dense blocks and three transition layers. Dense block (1) was constructed using a BN layer, ReLU, 3 × 1 × 1 3D convolution layer, BN layer, ReLU, and 3 × 3 × 3 3D convolution layer (Figure 5b). The structures of dense blocks (2), (3), and (4) are similar to that of dense block (1). Figure 5c shows the transition layer composed of a BN layer, ReLU, 3 × 1 × 1 3D convolution layer, and 2 × 2 × 2 average 3D pooling layer.

### 3.3. MLFF 3D CNN

Currently, outstanding performance has been achieved for image classification problems using different CNN models. An example is the fusing of multiple CNNs for feature aggregation, where extracting various spatial and temporal features is possible by creating different structures and depths [30]. Different convolutional layers can extract features at various levels of abstraction for the MLFF 3D CNN training phase. Various features can also be derived from this training process with varying depths and filters of different sizes. Using this approach, some of the related features lost in the layered architecture can be selected, rendering the final feature richer.

**Figure 6:**
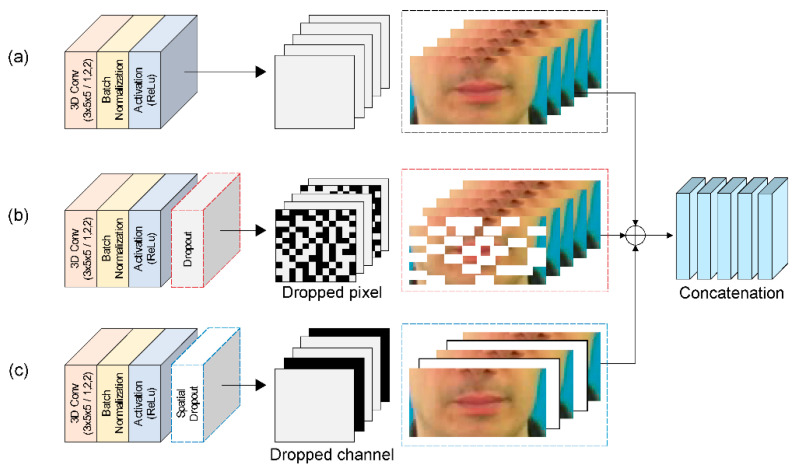
Detailed MLFF 3D CNN: (**a**) first architecture; (**b**) second architecture with dropout layer using dropped pixel; and (**c**) third architecture with spatial dropout layer using dropped channel.

The proposed MLFF 3D CNN architecture is shown in Figure 6. The first module (Figure 6a) consists of a 3D convolutional layer with 64 3D kernels of size 3 × 5 × 5 on a 3D densely connected convolution layer output feature, followed by a BN layer and ReLU layer. For the second module (Figure 6b), the structure of the first module is followed by a dropout layer to alleviate overfitting as the benchmark dataset used is not large compared with existing image datasets. The role of the dropout layer is to improve and generalize the performance by preventing the creation of strongly correlated activations, which solves overtraining and overfitting [31]. In the third module, the structure is similar to that of the second module, except that the dropout layer is replaced by a spatial dropout layer (Figure 6c), which is a method of dropping the entire feature map. Unlike the standard dropout method, which randomly drops pixels, this method exhibits excellent image classification using CNN models with strong spatial correlation [32]. Therefore, we applied a spatial dropout layer to effectively extract the shapes of the lips, teeth, and tongue with a strong spatial correlation that includes fine movements.

### 3.4. GRU

The GRU is a recurrent neural network that learns to propagate and regulate the flow of information over more time stages [33]. The GRU can distinguish longer temporal contexts, which is helpful for discriminating ambiguity because 3D CNN captures only short viseme-level features. Moreover, the gradient vanishing problem can be solved by using a GRU, which uses an update gate and reset gate.

**Figure 7:**
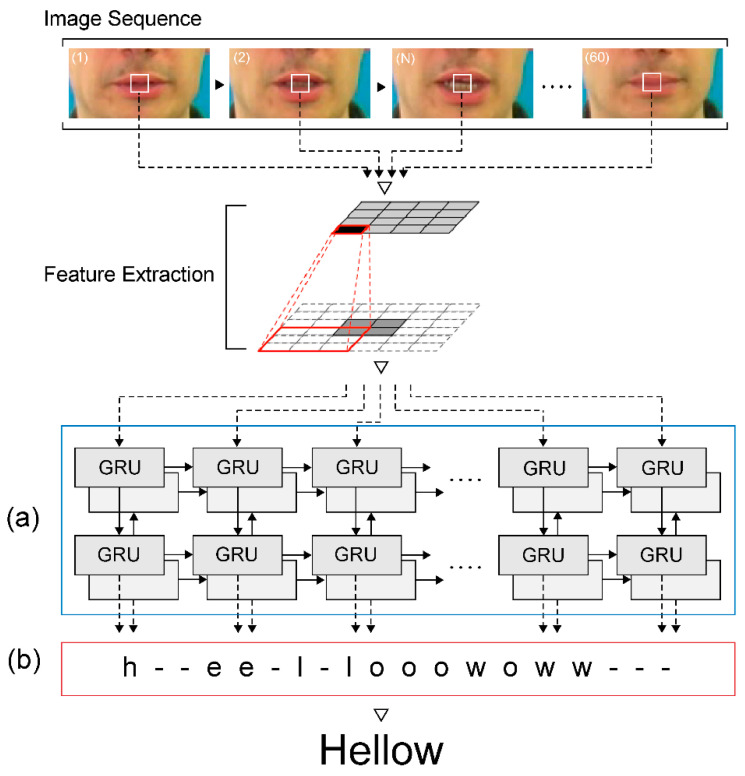
Sequence processing module: (**a**) a two-layer bi-directional GRU and (**b**) CTC.

Our proposed architecture uses a two-layer bi-directional GRU as a sequence processing module (Figure 7a). Unlike the typical deployment of GRU, a two-layer bi-directional GRU is employed to present information in both forward and backward manners to two separate neural network architectures that are connected to the same output layer, such that both networks can obtain complete information regarding the input. The two-layer bi-directional GRU layer receives its input from the MLFF 3D CNN sequentially and then generates characters as output, as follows:

(3) $$z_{t}=\sigma \left(W_{z}a_{t}+U_{z}h_{t-1}+b_{z}\right)$$

(4) $$r_{t}=\sigma \left(W_{r}a_{t}+U_{r}h_{t-1}+b_{r}\right)$$

(5) $$h_{t}=(1-z_{t})\;○h_{t-1}+z_{t}○\sigma_{r}\left(W_{h}a_{t}+U_{h}\left(r_{t}○h_{t-1}\right)+b_{h}\right)$$

The GRU consists of four components ($x_{t}$,$\;z_{t}$,$\;r_{t}$, and $h_{t}$) and a given sequence of image features a = ($a_{1},\;a_{2},\cdots$, $a_{t}$). $x_{t}$ is an input vector with its resulting weight parameter matrix and vector. $z_{t}$ is an update gate vector with its resulting weight parameter matrix and vectors $W_{z}$, $U_{z}$, and $b_{z}$. $r_{t}$ is a reset gate vector with its resulting weight parameter matrix and vectors $W_{r}$, $U_{r}$, and $b_{r}$. Finally, $h_{t}$ is an output vector with its resulting weight parameter matrix and vectors $W_{h}$, $U_{h}$, and $b_{h}$. $h_{t-1}$ is the previously hidden state output, which has the same structure as the current state. σ is the ReLU function, used as an activation function. ○ represents the Hadamard product. To obtain an output with 75 × 512 tensors using the merge layer, we provided an input sequence of 75 × 3 × 1 × 192 (time/width/height/channel) frames in a bi-directional GRU.

### 3.5. Connectionist Temporal Classification (CTC)

We used the CTC approach and applied it to an end-to-end deep neural network. This approach uses a loss function to parameterize the distribution of a label token sequence without having to align the input sequence [34]. A single set of time step label tokens can be expressed as V by using CTC, where the series of size-T given by the temporal module is the output labeled by the blank symbols ‘⊔ and consecutive symbols are repeated (Figure 7b). We define a function $Β\;:{\left(V\;\cup^{\;}\left\{⊔\right\}\right)}^{*}\to \;V^{*}$ to delete adjacent characters and to remove any blanks because the processed string may contain a blank token. It is possible to obtain the probability of observing a labeled sequence y by marginalizing this label, $p(y|x)=\sum_{u\in {Β}^{-1}\left(y\right)}p(u_{1}|x)\cdots p(u_{T}|x)$, where x is the input video, for all possible alignments. The standard CTC loss $L_{ctc}$ formula is defined as follows:

(6) $$p_{ctc}\left(y|x\right)=\underset{w\in B^{-1}\left(y\right)}{\sum }p_{ctc}\left(w|x\right)=\underset{w\in B^{-1}\left(y\right)}{\sum }\overset{T}{\underset{t=1}{\prod }}q_{w_{t}}^{t}$$

(7) $$L_{ctc}=-\ln\;p_{ctc}\left(y|x\right)$$

where T is the duration of the input sequence and $q_{\left(w_{t}\right)}^{t}$ represents the SoftMax probability of the output label $w_{t}$, where $w_{t}\in \left\{a,\;ai,\;an,\;ao,\cdots ,\;zun,\;zuo,\;blank\right\}$ at frame t. The CTC path of a sequence is $w=\left(w_{1},\;w_{2},\cdots ,\;w_{T}\right)$, and y is the sentence label (ground truth). $B^{-1}\left(y\right)$ is the set of all possible paths of the CTC that can be mapped to ground truth y. As CTC prohibits the use of autoregressive connections to control the inter-time-step dependencies of the label sequence, it is conditionally independent of the marginal distributions generated at each time step of the temporal module. Therefore, CTC models are typically decoded using a beam search procedure to restore the temporal dependency of the labels, which blends the probabilities of that language model.

## 4. Experiments and Results

This section describes the used dataset, data pre-processing, data augmentation, and implementation.

### 4.1. Dataset

The GRID audio-visual dataset is widely used for audio-visual recognition and VSR studies and is built on sentence-level audio video clips [16]. GRID is an openly available corpus containing an audio-visual database from 34 speakers with 1000 utterances per speaker and a total duration of 28 h. One sample has a single speaker clip, and each sample in this database lasts 3 s at 25 frames/s. The visual data for speaker number 21 are missing from the online available database corpus [16]. This database is sentence-level with a fixed grammar and is composed of “command (4) + color (4) + preposition (4) + letter (26) + digit (10) + adverb (4)”. It has 51 unique words, and each sentence is a randomly chosen combination of these words.

To unify the test conditions for our experiment, we divided the training and validation sets as follows [21]. In unseen-speaker datasets that were not historically used in the literature, 3971 videos were used for the evaluation data relevant to male speakers (1 and 2) and female speakers (20 and 22). The remaining videos for the unseen speakers (28,775 videos) were utilized to train the models. Following this strategy, the models were evaluated using speakers that had not appeared in the training process, thus guaranteeing the generalized performance of the model. We employed sentence-level variants of segmentation for overlapped-speaker datasets, where 255 random sentences from each speaker were used for evaluation. For training, the leftover data from all speakers were pooled.

### 4.2. Data Pre-Processing and Augmentation

The data pre-processing stage detects the targeted face and mouth using a DLib face detector [35]. This detector utilized a HoG feature-based linear classifier [35]. The output is given as the (*x*, *y*) coordinates of the diagonal edges; these coordinates are used later to draw the bounding box around the mouth. Subsequently, the iBug tool was used with 68 landmarks coupled with an online Kalman filter as a face landmark predictor [36]. This tool is typically used to read lip movements and extract points on the lips, which correspond to those obtained from the trained dataset. These tools were employed to extract a mouth-centered area with dimensions of 100 × 50 pixels per frame using an affine transformation and to standardize the RGB channels over the entire training set to have zero mean and unit variance. For training data, we used the data augmentation process from [21] to prevent overfitting. We performed training with both regular and horizontally mirrored image sequences. As the dataset included start and end terms that acted as a timer for each “clip” sample, we augmented the training data at the sentence level using individual words as additional training instances. These instances had a decay rate of 0.925. Finally, if necessary, we detected the movement speed and duplicated the frames to prevent variation, and this process was conducted with a probability of 0.05/frame. All models were trained and tested under the same pre-processing and augmentation processes for the GRID dataset.

### 4.3. Implementation

All models were implemented using Keras with a TensorFlow backend and TensorFlow-CTC decoder to measure the character error rate (CER) and word error rate (WER) scores using CTC beam search. In Figure 3 and Table A1 (Appendix A), the detailed configuration and parameters used for each layer in the proposed architecture are summarized. The network parameters of all models were initialized via He initialization, except for the orthogonally initialized square GRU matrices and the default hyperparameters. The orthogonally initialized square GRU matrices were trained with mini batches of size 8 and used the optimizer ADAM [37] with a learning rate of 0.0001. The proposed model was trained utilizing channel-wise dropped pixels and the dropped channel using spatial dropout in the MLFF 3D CNN, where the proposed models included the baseline model trained on GRID until overfitting.

As mentioned earlier, LipNet is the baseline model in our study; therefore, we evaluated its performance for the categories of unseen and overlapped speakers. For the unseen-speaker category, 8.534% CER and 16.341% WER were achieved, versus 6.400% CER and 11.400% WER in the model paper [21]. In addition, for overlapped speakers, we obtained CER of 5.657% and WER of 14.779%, whereas 1.900% CER and 4.800% WER were mentioned in the model paper: our test results for the LipNet model are higher than those of the original LipNet model. Due to these variations in outcomes, video clips of individual words were not used for additional training instances or other defective operations in the training phase. As one of the contributions of this analysis is a feasibility test of the proposed model, to obtain the required CER and WER, we did not further subdivide the baseline model. However, we compared the results acquired in our environment with those obtained in the testing environments of the existing models to analyze both the existing models and the proposed model.

### 4.4. Performance Evaluation Metrics

We used standard automatic speech recognition evaluation metrics to assess the proposed model. The learning loss of all the models was measured to evaluate the learning state during the training process. To compare the performances and computational efficiencies of all models, we evaluated the parameters, epoch time, CER, and WER, of each model.

By calculating the total edit distance, the error rate metrics used for accuracy assessment were obtained and converted into percentages. It is necessary to compare the decoded text to the original text when assessing misclassifications. The equation is given, wherein N is the cumulative number of characters in the ground truth, S is the number of characters substituted for incorrect classifications, I is the number of characters inserted for non-picked characters, and D is the number of deletions that should not be present for decoded characters. Thus, the CER and WER are determined using Equations (8) and (9), where C and W denote characters and words, respectively.

(8) $$\mathrm{CER}\left(\%\right)=\left(\frac{C_{S}+C_{D}+C_{I}}{C_{N}}\right)\times 100$$

(9) $$\mathrm{WER}\left(\%\right)=\left(\frac{W_{S}+W_{D}+W_{I}}{W_{N}}\right)\times 100$$

We performed a CTC beam search using a TensorFlow-CTC decoder implementation to generate approximate maximum-probability predictions for all experimental models. We also compared the CER and WER with respect to the number of parameters and computational efficiency over the epoch time. To visualize the results, we used the phoneme-to-viseme mapping described in [38].

### 4.5. Training Process and Learning Loss

**Figure 8:**
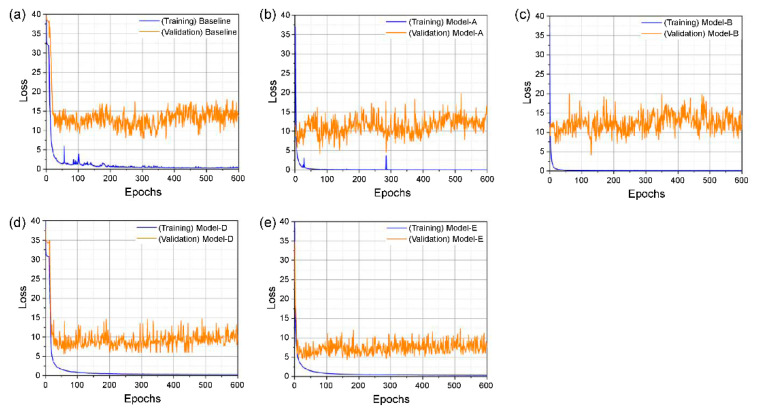
Training and validation loss of unseen speakers: (**a**) baseline; (**b**) model-A; (**c**) model-B; (**d**) model-D; (**e**) model-E.

**Figure 9:**
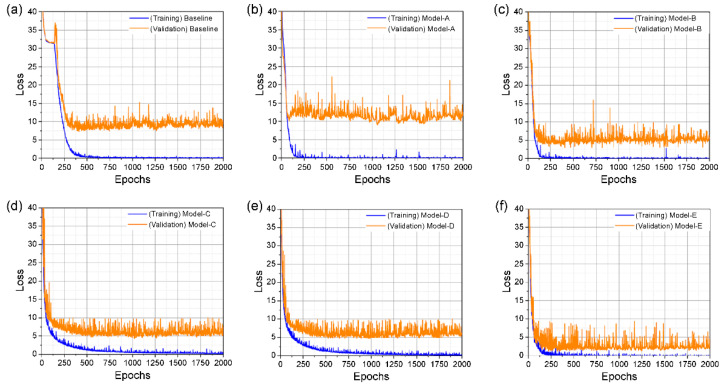
Training and validation loss of overlapped speakers: (**a**) baseline; (**b**) model-A; (**c**) model-B; (**d**) model-eC; (**e**) model-D; (**f**) model-E.

The training and validation losses during training on the GRID corpus are shown in Figure 8 and Figure 9, and the definition of each tested model is presented in Table 1 for both the unseen and overlapped-speaker categories. For the former category in Figure 8, the gaps between training and validation in models A and B and the baseline model are similar, as illustrated in Figure 8a–c. Furthermore, our proposed model shows large gaps relative to those for the three models in Figure 8a–c, but slightly smaller gaps relative to those in Figure 8d.

In the category of overlapped speakers, the training loss of each model started to decrease earlier than the baseline, as shown in Figure 9. The validation loss of the baseline and Figure 9b show similar tendencies, where the differences between the training and validation losses in Figure 9c–e are lower than the difference in the baseline model.

We performed the training process with the 3D ResNet-50 model; however, it did not perform as our experimental environment ran out of memory, and the results for unseen speakers were excluded. Finally, these results indicate that our proposed model for the two categories in the GRID dataset shows the lowest difference between the training and validation losses, which effectively prevents overfitting.

### 4.6. WER and CER

The results are reported for the unseen and overlapped speakers of the GRID dataset. The results of the comparison between our proposed model and the existing deep learning models are presented in Table 2, which reveals that our proposed model achieved state-of-the-art (SOTA) results: 2.853% CER and 5.059% WER for the unseen-speaker category and 1.004% CER and 1.011% WER for the overlapped-speaker category. These results exhibit decrease of all conditions over the current SOTA and baseline results.

**Figure 10:**
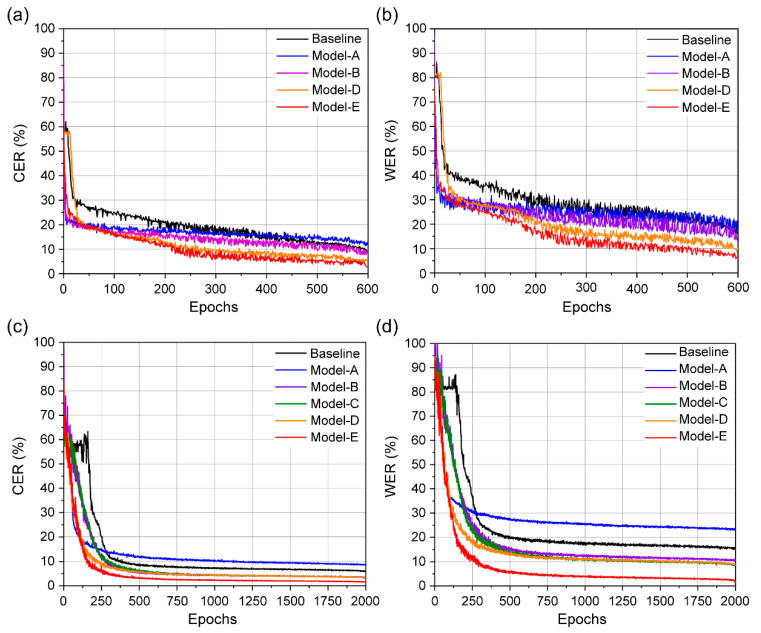
Training steps for CER and WER comparing our proposed model against the baseline and other models: (**a**) CER and (**b**) WER evaluated using unseen speakers, and (**c**) CER and (**d**) WER evaluated using overlapped speakers.

Although the accuracies of the models with 3D ResNet-50 and 3D DenseNet-121 architectures exceeded that of the baseline, no significant differences were detected (Table 2). In the case of unseen speaker′s category (Figure 10a,b), Models A, B, D, and E exhibit an almost steady learning behavior until approximately 250 epochs; subsequently, the error rates show a continuous decrement. On the other hand, both baseline and Model A with a simple structure show higher error rates compared to others. Moreover, the smallest error rates are achieved by our proposed Model E with the same training steps. For the overlapped speaker category, Models B, C, and D show similar performance. Unlike the comparable performance of Model A for the unseen speaker category, an obvious degradation in performance is observed for the same model in the case of overlapped speaker’s category (Figure 10c,d). For our proposed Model E, the error rate shows a noticeable decrement after approximately 130 epochs. Therefore, in terms of accuracy, our proposed model outperforms the existing models, including the baseline model, which can be attributed to the combination of multiple 3D CNN architectures. Figure 10 shows the training step with CER and WER on the GRID database.

### 4.7. Model and Computational Efficiency

**Figure 11:**
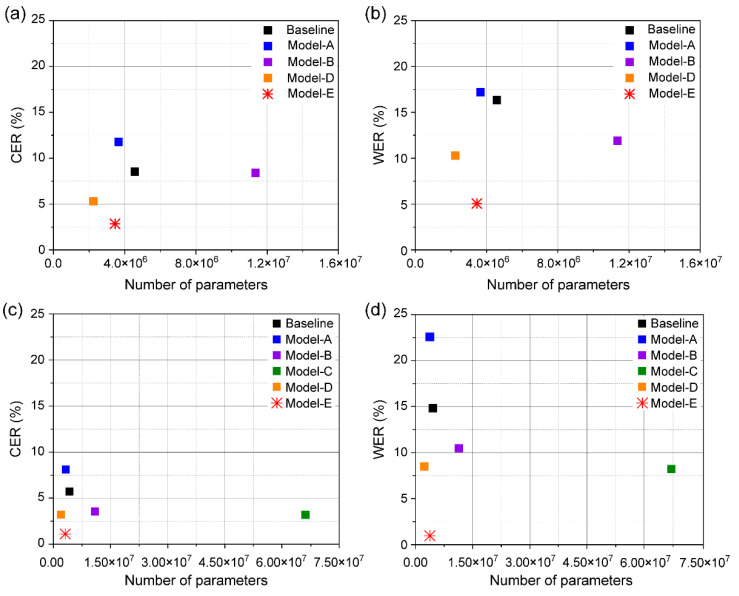
CER and WER of the baseline and different models according to the number of parameters, measured on two splits: (**a**) CER and (**b**) WER evaluated using unseen speakers, and (**c**) CER and (**d**) WER evaluated using overlapped speakers.

**Figure 12:**
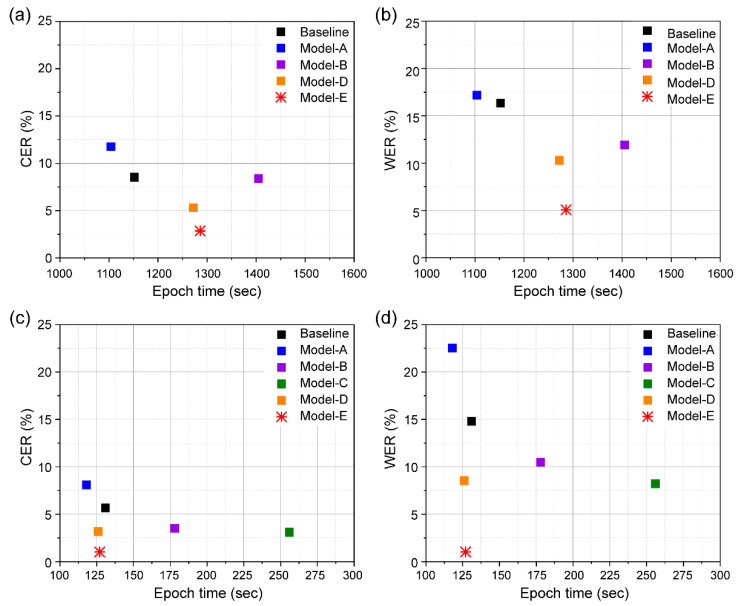
CER and WER of the baseline and different models according to epoch time, measured on two splits: (**a**) CER and (**b**) WER evaluated using unseen speakers, and (**c**) CER and (**d**) WER evaluated using overlapped speakers.

The major limitations of VSR systems in practical applications are their model size and computational efficiency. To evaluate the computational efficiencies of the models, we compared their accuracies with different numbers of trained parameters and epoch times (Figure 11 and Figure 12). We summarized the number of parameters and epoch time for individual models for the two dataset categories in Table 1. Although our proposed model has an epoch time similar to that of the baseline model, lower CER and WER are seen due to 10 M fewer parameters used. In addition, our model shows a faster epoch time and lower number of parameters than those of the three other models except Model A with the GRID dataset. LeNet-5 has a gradient-based learning CNN structure, which is divided into an input layer, a convolution layer, a pooling layer, a fully connected layer, and an output layer, and the input layer is removed, and a total of seven layers are included. Model A is a simple structure using two convolution layers and pooling layers from LeNet-5. This simple structure has difficulty in processing high resolution images. Therefore, its accuracy will be significantly lowered when used for an application like lip reading, which requires delicate motion detection.

In conclusion, we obtained a lower error of GRID database while reducing the number of parameters by approximately 11.2 M compared to that of the baseline model; we also achieved a comparable epoch time.

### 4.8. Confusion Matrix

**Figure 13:**
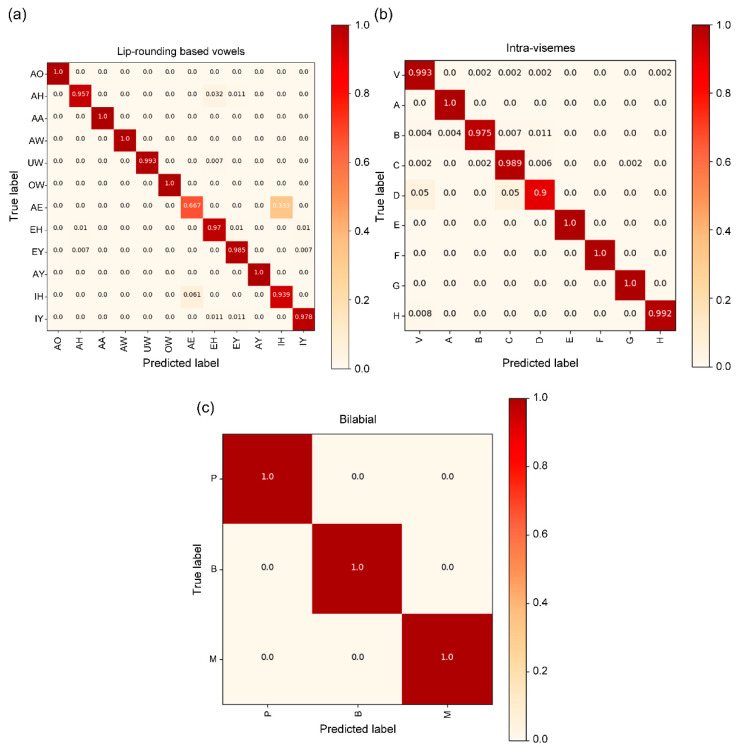
Detailed proposed architecture confusion matrices for the (**a**) lip-rounding based vowels; (**b**) intra-visemes; and (**c**) bilabial groups. The three groups with the most confusions were selected, as well as the confusions within viseme clusters.

A mapping proposed by the IBM ViaVoice database was used for the visual analysis [38]. It consists of 43 phonemes grouped into 13 classes of visemes, including a silence class, vowels based on lip rounding (V), alveolar-semivowels (A), alveolar-fricatives (B), alveolar (C), palato-alveolar (D), bilabial (E), dental (F), labio-dental (G), and velar (H). For the bilabial viseme class, we plot a confusion matrix corresponding to the most confusing phoneme (Figure 13a), which represents the vowels based on lip rounding. In the experimental results, {/AE/, /IH/} is frequently misclassified during the text decoding process (Figure 13a). At a first glance, the confusion between /IH/ (a rather close vowel) and /AE/ (a very open vowel) is unexpected but only occurs in “at”, a generally pronounced feature word with a shortened, weak vowel /AH/ in sample /AE/. This effect is due to the similar pronunciations of the {at, bin} text pair. Figure 13b represents the intra-viseme categorical confusion matrix. Distinguishing homophones is a major challenge, and we present the experimental results for [p], [b], and [m] in Figure 13c. Based on the evaluation of our model from different aspects, this model can help overcome the technical barriers for the practical implementation of VSR systems.

The confusion matrix in [21] shows the results for frequent misclassifying words in the text decoding process. Many errors occurred, but {/AA/, /AY/} and {/AE/, /IH/} accounted for the largest proportion among them. Furthermore, similar pronunciation between the text pairs of {r, i}, {at, bin}, and {four, two} causes frequent incorrect classification during the text decoding process for {/AA/, /AY /}, {/AE/, /IH/} and {/AO/, /UW/} in [39]. Although it was difficult to distinguish words of similar pronunciation for {/AE/, /IH/} in our proposed model, an enhancement can be noticed in Figure 13a for correctly classifying similar pronunciations for {/AA/, /AY/} and {/AO/, /UW/}, unlike their frequent misclassification in [21,39]. Additionally, we can determine the superiority of our model performance by comparing it with the bilabial and intra-visemes categorical confusion matrix in [21,39]. Therefore, our model showed excellent performance in distinguishing all similar pronunciations on bilabial-visemes and intra-visemes.

## 5. Discussion and Conclusions

The primary reason lipreading is difficult is that much of the image in the video input remains unchanged—the movement of the lips is the biggest distinction. However, it is possible to perform action recognition, which is a type of video classification, from a single image. When lipreading, it is always important to derive the characteristics relevant to the speech content from a single image and to analyze the time relationship between the entire series of images to infer the content. The key problem with lipreading is visual ambiguity.

This paper presented a novel lipreading architecture for sentence-level VSR. By applying multiple visual feature extraction methods, we achieved accurate viseme prediction. To the best of our knowledge, this is the first time that a 3D CNN, 3D densely connected CNN, and MLFF 3D CNN have been used in combination to extract the features of lip movements as encoders. The strengths of each stage are as follows. The 3D CNN extracts features from multiple consecutive video frames efficiently. The 3D densely connected CNN helps in fully utilizing the features, effectively reducing the problem of gradient disappearance, and making the network deeper. In addition, the bottleneck layer, translation layer, and smaller growth rate make the network narrower, thereby reducing the number of model parameters, suppressing overfitting and saving computational power. Lastly, the MLFF 3D CNN with a dropout and spatial dropout layer avoids overfitting and effectively extracts shapes with strong spatial correlations with fine movements while exploring the context information of the movement in both the temporal and spatial domains.

We compared several deep learning models for predicting sentence sequences, and the results indicated that our proposed architecture achieves SOTA CER and WER values (Table 2). Smaller numbers of parameter and faster epoch times than those of the existing methods were realized using our proposed model. Moreover, the proposed architecture showed reduced CER and WER values than those of the baseline model for both the unseen-speaker and overlapped-speaker datasets.

It is important to develop a VSR system that exclusively uses visual information. This system has practical potential for various applications in speech recognition in noisy or underwater environments, biometric identification for security, and silent movie analysis; furthermore, it could be beneficial for patients with speech impairments. However, it remains difficult to perform automatic speech recognition using only VSR as speech uses acoustic and visual information. Thus, in future work, we will investigate a solution that can be directly applied to the loss function, because the loss function was not modified in our proposed model. Moreover, we intend to expand our concept to pursue performance enhancement and discover potential applications using both audio and visual information.

## Figures and Tables

**Table 1:** Number of parameters and epoch time of the proposed method compared to those of the baseline and different methods.

Model	Method		Parameters	Unseen Speakers	Overlapped Speakers
	Frontend	Backend		Epoch Time (s)	Epoch Time (s)
Baseline	3D CNN	Bi-GRU + CTC	45.7M	1152	131
Model-A	3D CNN + 3D LeNet-5	Bi-GRU + CTC	36.5M	1104	118
Model-B	3D CNN + 3D VGG-F	Bi-GRU + CTC	113.4M	1405	178
Model-C	3D CNN + 3D ResNet-50	Bi-GRU + CTC	667M	-	256
Model-D	3D CNN + 3D DenseNet-121	Bi-GRU + CTC	22.4M	1272	126
Model-E	Proposed architecture		34.5M	1286	127

**Table 2:** Performance of the proposed model compared to the baseline model and different existing models with unseen and overlapped speakers.

Year	Model		Unseen Speakers		Overlapped Speakers	
			CER (%)	WER (%)	CER (%)	WER (%)
	Hearing-impaired person (avg.) [21]		-	47.700	-	-
2016	Baseline-LSTM [21]		38.400	52.800	15.200	26.300
2016	Baseline-2D [21]		16.200	26.700	4.300	11.600
2017	LipNet-NoLM [21]		-	-	2.000	5.600
2017	LipNet [21]		6.400	11.400	1.900	4.800
2017	WAS [5]		-	-	-	3.300
2018	LCANet [39]		-	-	1.300	2.900
2018	LipNet + 3D-FPA [9]		7.246	14.178	-	-
2019	LRNeuNet [40]		6.100	9.500	1.200	2.700
2019	LipSound [41]		-	-	1.532	4.215
2020	PCPG [42]		-	11.200	-	-
2020	FastLR [43]		-	-	2.400	4.500
2020	LipNet + LipsID [44]		5.200	9.900	1.200	3.300
2020	TVSR-Net + SC-Block [45]		-	90.900	-	-
2020	DualLip [46]		-	-	1.600	2.710
2021	3D-ResNet50-TCN-CTC [47]		4.100	6.200	1.200	1.100
	Frontend	Backend	CER (%)	WER (%)	CER (%)	WER (%)
Baseline	3D CNN	Bi-GRU + CTC	8.534	16.341	5.657	14.779
Model-A	3D CNN + 3D LeNet-5	Bi-GRU + CTC	11.797	17.188	8.083	22.526
Model-B	3D CNN + 3D VGG-F	Bi-GRU + CTC	8.395	11.914	3.499	10.482
Model-C	3D CNN + 3D ResNet-50	Bi-GRU + CTC	-	-	3.089	8.203
Model-D	3D CNN + 3D DenseNet-121	Bi-GRU + CTC	5.314	10.286	3.165	8.529
Model-E	Proposed architecture		2.853	5.059	1.004	1.011

## Data Availability

Not applicable.

## Appendix A

The following table provides a detailed hyperparameters further describing the proposed end-to-end lip-reading architecture.

**Table A1:** Hyperparameters of proposed architecture.

Layers	Size/Strid/Pad		Output Size	Dimension Order
3D Conv	[3 × 5 × 5]/(1, 2, 2)/(1, 2, 2)		75 × 50 × 25 × 64	T × C × H × W
3D Max Pooling	[1 × 2 × 2]/(1, 2, 2)		75 × 50 × 13 × 64	T × C × H × W
3D Dense Block (1)	[3 × 1 × 1] 3D Conv	(×6)	75 × 25 × 13 × 96	T × C × H × W
	[3 × 3 × 3] 3D Conv			
3D Transition Block (1)	[3 × 1 × 1] 3D Conv		75 × 12 × 6 × 6	T × C × H × W
	[1 × 2 × 2] average pool/(1 × 2 × 2)			
3D Dense Block (2)	[3 × 1 × 1] 3D Conv	(×12)	75 × 12 × 6 × 38	T × C × H × W
	[3 × 3 × 3] 3D Conv			
3D Transition Block (2)	[3 × 1 × 1] 3D Conv		75 × 6 × 3 × 3	T × C × H × W
	[1 × 2 × 2] average pool/(1 × 2 × 2)			
3D Dense Block (3)	[3 × 1 × 1] 3D Conv	(×24)	75 × 12 × 6 × 38	T × C × H × W
	[3 × 3 × 3] 3D Conv			
3D Transition Block (3)	[3 × 1 × 1] 3D Conv		75 × 3 × 1 × 1	T × C × H × W
	[1 × 2 × 2] average pool/(1 × 2 × 2)			
3D Dense Block (4)	[3 × 1 × 1] 3D Conv	(×16)	75 × 3 × 1 × 33	T × C × H × W
	[3 × 3 × 3] 3D Conv			
MLFF 3D CNN (1)	[3 × 5 × 5]/(1, 2, 2)/(1, 2, 2)		75 × 3 × 1 × 64	T × C × H × W
MLFF 3D CNN (2)	[3 × 5 × 5]/(1, 2, 2)/(1, 2, 2)		75 × 3 × 1 × 64	T × C × H × W
MLFF 3D CNN (3)	[3 × 5 × 5]/(1, 2, 2)/(1, 2, 2)		75 × 3 × 1 × 64	T × C × H × W
Bi-GRU (1)	256		75 × 512	T × F
Bi-GRU (2)	256		75 × 512	T × F
Linear	27 + blank		75 × 512	T × F
Softmax			75 × 28	T × V
